# Development of a generic *Ehrlichia* FRET-qPCR and investigation of ehrlichioses in domestic ruminants on five Caribbean islands

**DOI:** 10.1186/s13071-015-1118-5

**Published:** 2015-10-06

**Authors:** Jilei Zhang, Patrick Kelly, Weina Guo, Chuanling Xu, Lanjing Wei, Frans Jongejan, Amanda Loftis, Chengming Wang

**Affiliations:** Jiangsu Co-innovation Center for Prevention and Control of Important Animal Infectious Diseases and Zoonoses, Yangzhou University College of Veterinary Medicine, Yangzhou, Jiangsu 225009 P. R. China; Ross University School of Veterinary Medicine, Basseterre, St. Kitts and Nevis; Anhui Science and Technology University College of Animal Science, Anhui, China; Utrecht Centre for Tick-borne Diseases (UCTD), FAO Reference Centre for Ticks and Tick-borne Diseases, Faculty of Veterinary Medicine, Utrecht University, Yalelaan 1, 3584 CL Utrecht, The Netherlands; Department of Veterinary Tropical Diseases, Faculty of Veterinary Science, University of Pretoria, Private Bag X04, Onderstepoort, 0110 South Africa

**Keywords:** *Ehrlichia*, FRET-qPCR, Domestic ruminants, Caribbean

## Abstract

**Background:**

The *Ehrlichia* are obligate intracellular Gram-negative tick-borne bacteria that are important human and animal pathogens. There is a need for assays to rapidly and reliably detect and differentiate the five generally recognized species into groups in a single reaction: *E. canis*, *E. chaffeensis*, *E. ewingii*, *E. muris* and *E. ruminantium*.

**Methods:**

We developed primers and probes against the *16S rRNA* gene to enable us to reliably detect the five major *Ehrlichia* spp. in a single FRET-qPCR. We tested the *Ehrlichia* FRET-qPCR on reference strains and on DNA from the blood of domestic ruminants from five Caribbean islands. The *Ehrlichia* present were determined using melting point analysis and by sequencing the *Ehrlichia* FRET-qPCR products as well as those of a nested PCR against the citrate synthase gene (*gltA*).

**Results:**

Our *Ehrlichia* FRET-qPCR was negative for the closely related *Anaplasma marginale* and *A. phagocytophilum* but gave positive reactions with reference strains of the most generally recognized species and with other less characterized *Ehrlichia* of domestic ruminants, mainly *E. ovina*, the Panola Mountain *Ehrlichia*, and *Ehrlichia* sp. BOV2010. Melting point analysis revealed 4 distinct groups: *E. ruminantium* (*T*_m_ ~55.8 °C); *E. chaffeensis* and *E. ewingii* (*T*_m_ ~57.7 °C); *E. canis*, *E. muris*, *E. ovina* and *Ehrlichia* sp. BOV 2010 (*T*_m_ ~62.0 °C); and the Panola Mountain *Ehrlichia* (*T*_m_ ~65.5 °C). The detection limit of the FRET-qPCR was ~ 5 gene copies in a reaction and the sequences of the FRET-qPCR products were as expected. With DNA from domestic ruminants from the Caribbean we found 12.2 % (134/1,101) positive: cattle (76/385; 19.7 %), sheep (45/340; 13.2 %) and goats (13/376; 3.5 %). Melting point analysis and sequencing of the FRET-qPCR and nested PCR *gltA* products showed the *Ehrlichia* we detected were *E. canis* or very closely related organisms.

**Conclusions:**

In a single reaction, our *Ehrlichia* FRET-qPCR can detect the *Ehrlichia* spp. we studied and differentiate them into four groups. Domestic ruminants in the Caribbean are not uncommonly exposed to *Ehrlichia*, possibly *E. canis* or very closely related organisms.

## Background

*Ehrlichia* are obligate intracellular Gram-negative tick-borne bacteria that are important animal and human pathogens. There are five generally recognized *Ehrlichia* spp., mainly *E. canis*, *E. chaffeensis*, *E. ewingii*, *E. muris* and *E. ruminantium* [1, 2]. *Ehrlichia ruminantium* is the most important in domestic ruminants, where it causes heartwater, an acute disease associated with very high mortality (up to 90 %) and extensive economic losses [3]. Although various serological tests for *E. ruminantium* have been described, in particular ELISAs detecting antibodies to the organism’s major antigenic protein (MAP), inappropriate positive results are not uncommon, probably due to cross-reactivity with other tick-borne *Ehrlichia* spp. [4–10]. A number of such *Ehrlichia* that might be responsible for the serological cross-reactivity have been described in domestic ruminants, including *E. ovina* in a sheep from Turkey [4], *E. chaffeensis* in goats and cattle in the USA [11, 12], the Panola Mountain *Ehrlichia* in goats in the USA [9], and *Ehrlichia* sp. BOV2010/*Ehrlichia* sp. UFMT-BV in cattle in the Americas [13, 14]. There are also other *Ehrlichia* that have been reported in domestic ruminants, but stocks are not readily available and their taxonomic status is yet to be confirmed [15]. These include *E. ondiri* [15], *Ehrlichia* sp. Omatjenne [16], *Ehrlichia* sp. Germishuys [16] and an *Ehrlichia* sp. from Zimbabwe [7].

In a recent study in the Caribbean, inappropriate positive MAP-1B ELISA results for *E. ruminantium* were reported for domestic ruminants from four of the seven islands studied [10]. These inappropriate positive reactions were thought to be due to infections with other *Ehrlichia* spp., and the presence of these organisms made serological testing for *E. ruminantium* unreliable in the Caribbean, as has been shown to be the case in Africa [17]. Being able to reliably detect *E. ruminantium* is important as it is not only a serious threat to local livestock production, but also to animals on the American mainland [18]. To further investigate ehrlichioses in domestic ruminants in the Caribbean, we developed a generic *Ehrlichia* FRET-qPCR that would enable us in a single reaction to specifically and reliably detect the major *Ehrlichia* spp. and differentiate them into groups. The development and validation of this PCR and its use to screen domestic ruminants in the Caribbean for *Ehrlichia* spp. is described below.

## Methods

### Blood samples

Whole blood samples (*n* = 1,101) in EDTA were collected from apparently healthy domestic ruminants (cattle, sheep and goats) on Montserrat (*n* = 77), St. Kitts (*n* = 373), Grenada (*n* = 140), Nevis (*n* = 262) and Dominica (*n* = 249) as described previously [10, 19] (Table 1). Aliquots of 200 μl were frozen at −20 °C until DNA was extracted for PCR studies. Ethical Approval: All work in this study was reviewed and approved by the Institutional Animal Care and Use Committee of Ross University School of Veterinary Medicine. Owners of the animals provided consent for blood samples to be collected.

**Table 1:** Domestic ruminants from five Caribbean islands found positive in the generic Ehrlichia FRET-qPCR

Island	Positive animals % (positive/total N)		
	All animals	Individual animal species	
Montserrat	20.8 % (16/77)	Goat	15.8 % (3/19)
		Sheep	26.1 % (12/46)
		Cattle	8.3 % (1/12)
St. Kitts	25.5 % (95/373)	Goat	3.4 % (1/29)
		Sheep	31.3 % (31/99)
		Cattle	25.7 % (63/245)
Grenada	3.6 % (5/140)	Goat	5.1 % (4/79)
		Sheep	1.6 % (1/61)
Nevis	0.38 % (1/262)	Goat	0.7 % (1/137)
		Sheep	0.0 % (0/82)
		Cattle	0.0 % (0/43)
Dominica	6.8 % (17/249)	Goat	0.36 % (4/112)
		Sheep	1.9 % (1/52)
		Cattle	14.1 % (12/85)

### *Ehrlichia* strains

As positive controls we used the five major *Ehrlichia* spp., mainly *E. ruminantium*, *E. canis*, *E. chaffeensis*, *E. ewingii* and *E. muris.* We also tested *Ehrlichia* that were available to us and have been previously reported to occur in ruminants, mainly *E. ovina* [4], *Ehrlichia* sp. BOV2010 [13] and the Panola Mountain *Ehrlichia* [9]. We used DNA extracted in previous studies from *E. ruminantium* [10] and *E. canis* [20], DNA extracted as described below from tissue cultures of *E. canis* (Oklahoma) and *E. chaffeensis* (Arkansas) (supplied by Gregory Dasch, Centers for Disease Control, Atlanta), from blood stabilates (*E. ovina* and *Ehrlichia* sp. BOV2010), and from an *Amblyomma variegatum* positive for the Panola Mountain *Ehrlichia* by PCR (unpublished data). We also used plasmids that were created to contain an appropriate portion of the *16S rRNA* gene of *E. ewingii* and *E. muris* using the pIDTSMART cloning vector (Integrated DNA Technologies, Coralville, IA, USA) and linearization with *Hin*dIII (Promega, Madison, WI, USA).

To test the specificity of our PCR, we tested DNAs extracted from blood of cattle verified to be infected with *A. marginale* (identical nucleotide *16S rRNA* sequences with CP006847) and *A. phagocytophilum* (identical *16S rRNA* sequences with KJ782389).

### DNA extraction

The High-Pure PCR Template Preparation Kit (Roche Molecular Biochemicals, Indianapolis, IN, USA) was used according to the manufacturer’s instructions to extract total nucleic acids from the samples (200 μl). The extracted DNAs were eluted in 200 μl elution buffer and stored at −80 °C.

### Development of a generic *Ehrlichia* FRET-qPCR

#### Primers and probes

**Fig. 1:**
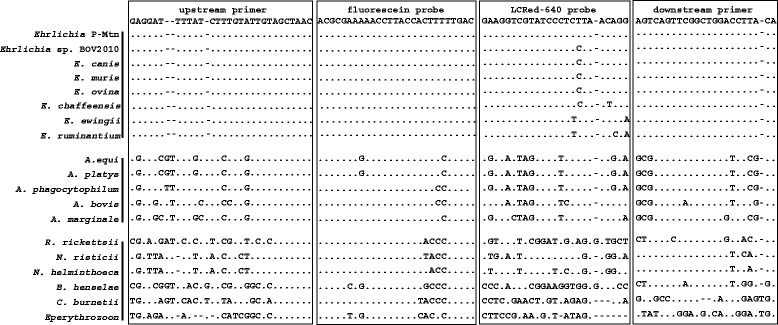
Alignment of the primers and probes of the generic *Ehrlichia* FRET-qPCR with the *16S rRNA* gene sequences of *Ehrlichia* spp. and related genera/species. The sequences of the upstream/downstream primers and the fluorescein/LCRed 640 probes are shown at the top of the boxes. The upstream primer and two probes were used as the indicated sequences while the downstream primer was used as an antisense oligonucleotide. The 6-FAM label was attached directly to the 3-terminal nucleotide of the fluorescein probe and the LCRed 640 fluorescein label was added via a linker to the 5′-end of the LCRed 640 probe. Dots indicate nucleotides identical to the primers and probes, and dashes denote the deletion of a nucleotide. Both of the primers and the fluorescein probe had 100 % identity with all *Ehrlichia* spp. while the LCRed probe had 0, 1, 2 or 3 nucleotide mismatches. The primers and probes had multiple mismatches with other related organisms

The *16S rRNA* sequences for the five major *Ehrlichia* spp. and those reliably reported in domestic ruminants, five *Anaplasma* spp., and six related bacteria were obtained from GenBank: *E. canis* (EU178797, GU810149), *E. ruminantium* (CR925678, DQ647616, U03776, U03777), *E. chaffeensis* (AF147752, U60476), *E. ewingii* (M73227, U96436), *E. muris* (AB013008, AB196302), *E. ovina* (AF318946), *Ehrlichia* sp. BOV 2010 (HM486680), the Panola Mountain *Ehrlichia* (DQ324367); *A. equi* (AF172167), *A. platys* (M82801), *A. phagocytophilum* (AY055469), *A. bovis* (HQ913646), *A. marginale* (AF309866, AF414873); *Bartonella henselae* (AY513504); *Rickettsia rickettsii* (L36217), *Neorickettsia helminthoeca* (U12457), *Neorickettsia risticii* (NR029162); *Coxiella burnetii* (D89798), and *Eperythrozoon* sp. (FR869692) (Fig. 1). The sequences were aligned and regions were identified for primers and probes based on the conserved and variable areas of the alignments. The forward primer (5′-GAGGATTTTATCTTTGTATTGTAGCTAAC-3′), reverse primer (5′-TGTAAGGTCCAGCCGAACTGACT-3′) and fluorescein probe (5′-ACGCGAAAAACCTTACCACTTTTTGAC-6-FAM-3′) we selected had identical sequences in all the *Ehrlichia*. The LCRed 640 probe (5′-LCRred640-GAAGGTCGTATCCCTCTTAACAGG-phos-3′) was identical to the Panola Mountain *Ehrlichia* but had one nucleotide mismatch with *E. canis*, *E. muris, E. ovina* and *Ehrlichia* sp. BOV 2010*,* two mismatches with *E. ewingii* and *E. chaffeensis*, and three mismatches with *E. ruminantium* (Fig. 1). In contrast, the primers and probes had multiple mismatches (19–57) with *Anaplasma* spp. and other related bacteria (Fig. 1). When we used the BLAST to compare the primers and probes we developed against all sequences available on GenBank, we found they reliably detected the *Ehrlichia* spp. against which they were designed and that the nucleotide polymorphisms we used in the probes for the different species were highly conserved.

#### Thermal cycling and melting curve analysis

**Fig. 2:**
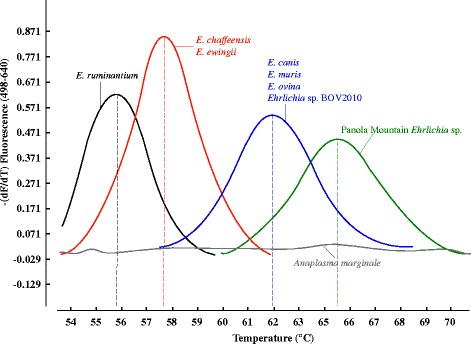
Composite of melting curves obtained with the generic *Ehrlichia* FRET-qPCR performed on various *Ehrlichia* species. The nucleotide mismatches between amplicons of the various species and the LCRed-640 probe we designed (Fig. 1) enabled us to distinguish four groups of *Ehrlichia* based on their previously determined *T*_m_: Panola Mountain *Ehrlichia* ~65.5 °C (green line); *E. canis*, *E. muris*, *E. ovina* and *Ehrlichia sp.* BOV2010/*Ehrlichia sp.* UMFG-EV ~62.0 °C (blue line); *E. chaffeensis* and *E. ewingii* (red line) ~57.6 °C; *E. ruminantium* ~55.8 °C (black line). No amplification peak was seen with *A. marginale* DNA (grey line)

High-resolution melting curve analysis following PCR was performed on a Roche Light-Cycler 480-II platform as described before [21, 22]. Each reaction was performed in a 20 μL final volume containing 10 μL of extracted DNA. Thermal cycling consisted of 1 activation cycle of 5 min at 95 °C followed by 45 fluorescence acquisition cycles consisting of 10 s at 95 °C, 15 s at 58 °C, and 15 s at 72 °C. Melting curve analysis was performed by monitoring fluorescence between 45 °C and 80 °C after 30 s at 95 °C. Data were analyzed as 640 nm: 530 nm (F4/F1) fluorescence ratios, and the first derivative of F4/F1 (−d(F4/F1)/dt) was evaluated (Fig. 2). The *T*_*m*_ value is influenced not only by nucleotide mismatches but also the types of nucleotides and GC percentage of the probes.

#### Sensitivity

For quantitative standards we used amplified DNA of *E. canis* identified in a previous study [20]. These *E. canis* DNA amplification products were confirmed by nucleotide sequencing (GenScript, Nanjing, Jiangsu, China) before being gel purified with a QIAquick Gel Extraction Kit (Qiagen, Valencia, CA) and quantified using the PicoGreen DNA fluorescence assay (Molecular Probes, Eugene, OR). The molarity of the *E. canis* DNA was estimated using the calculated molecular mass of the amplicons [23] and dilutions made to give solutions containing 10,000, 1,000, 100, 10, and 1 gene copies/μl in T_10_E_0.1_ buffer which were used as quantitative standards.

#### Specificity

The specificity of the positive control PCRs with the five widely recognized *Ehrlichia* spp. (DNAs of *E. canis*, *E. chaffeensis* and *E. ruminantium*, and plasmids representing *E. ewingii* and *E. muris*) were confirmed by electrophoresis of amplicons through 1.5 % MetaPhor agarose gels, purification using the QIAquick PCR Purification Kit (Qiagen, Valencia, CA, USA) and sequencing of both DNA strands using the appropriate forward and reverse primers (GenScript, Jiangsu, Nanjing, China). No reaction products were obtained when our generic *Ehrlichia* FRET-qPCR was performed with DNAs of *A. marginale* or *A. phagocytophilum*.

### Nested PCR for the citrate synthase gene of *Ehrlichia*

To amplify the citrate synthase gene (*gltA*) of *Ehrlichia* spp., we carried out nested PCRs (outside primers: EHRCS-131F and EHRCS-1226R, and inside primers: EHRCS-754F and EHRCS-879R, which amplify 1,108 and 126 bp sections of the gene, respectively) as described previously [9]. The PCR products we obtained were verified by gel electrophoresis, purified using the QIAquick PCR Purification Kit (Qiagen, Valencia, CA, USA) and sequenced (GenScript, Jiangsu, Nanjing, China).

## Results

### Development of a generic *Ehrlichia* FRET-qPCR

The generic *Ehrlichia* FRET-qPCR we established produced amplicons with each of the five well recognized *Ehrlichia* spp. that we tested, mainly *E. ruminantium*, *E. chaffeensis*, *E. ewingii, E. canis* and *E. muris*. The FRET-qPCR was also positive with DNA from *E. ovina*, the Panola Mountain *Ehrlichia*, and *Ehrlichia* sp. BOV 2010. Sequences of the amplification products were as expected for each organism (results not shown). No products were obtained when the generic *Ehrlichia* FRET-qPCR was performed with DNA from *A. marginal*e and *A. phagocytophilum* (results not shown). Further, we obtained no amplification products when we tested DNA from 60 of the cattle from St Kitts that were seropositive for *Anaplasma marginale* which is endemic and highly prevalent in the Caribbean (results not shown) (Kelly PJ, unpublished data).

Melting curve analysis enabled us to identify 4 distinct groups of *Ehrlichia* based on their *T*_m_: *E. ruminantium* ~55.8 °C; *E. chaffeensis* and *E. ewingii* ~57.7 °C; *E. canis*, *E. muris*, *E. ovina* and *Ehrlichia* sp. BOV 2010 ~ 62.0 °C; the Panola Mountain *Ehrlichia* ~65.5 °C (Fig. 2). No reaction products or melting peaks were found with the positive DNAs of *A. marginale* and *A. phagocytophilum*. When we tested around 300 copies of *E. ruminantium*, *E. chaffeensis*, *Ehrlichia* sp. BOV 2010 and the Panola Mountain *Ehrlichia*/μL in a single reaction, the *Ehrlichia* FRET-qPCR revealed 4 distinct melting curves with temperatures identical to those found with individual FRET-qPCRs of the agents.

With the quantitative standards developed using purified *E. canis* DNA, we determined that the detection limit of the generic *Ehrlichia* FRET-qPCR was ~5 copies of the *16S rRNA* gene per PCR.

### Prevalence of *Ehrlichia* spp. in domestic ruminants from five Caribbean islands

Of the 1,101 blood samples we examined, 134 (12.2 %) were positive for *Ehrlichia* spp. in our generic *Ehrlichia* FRET-qPCR (Table 1). Cattle were most commonly positive (19.7 %; 76/385), followed by sheep (13.2 %; 45/340) and goats (3.5 %; 13/376). The average *16S rRNA* copy number in the *Ehrlichia*-positive samples was 231 per μl of blood. All positive reactions had a *T*_m_ of ~62.0 °C and sequencing of seven animals’ *16S rRNA* amplicons showed the organisms we detected were 98–100 % identical with strains of *E. canis* from Turkey (Kutahya:AY621071) and the Philippines (D28A: JN121380), *Ehrlichia* sp. BOV2010 (HM486680) from Canada and the *Ehrlichia* UFMG-EV (JX629805) from Brazil [24]. They also had 98–100 % similarity with *E. ovina* and the *Ehrlichia* sp. Germishuys (U54805) which has a *16S rRNA* sequence 99.9 % identical to *E. canis* [16]. Sequencing of 15 of the nested *gltA* products we obtained revealed eight (Group 1; Table 2) had 98 % identity with the *Ehrlichia* sp. BOV2010 (JN673762) and the *Ehrlichia* sp. UFMG-EV (JX629807), 5 (Groups 3 and 4; Table 2) had 96 % similarity with *E. canis* from the US (Jake; NC007354) and Italy (AY647155), one (Group 5; Table 2) had 99 % identity with an *Ehrlichia* identified in a cattle tick in Africa (AF311965) [25], and one (Group 2; Table 2) had 95 % similarity with the *Ehrlichia* sp. BOV2010 (JN673762) and the *Ehrlichia* sp. UFMG-EV (JX629807). All the groups generally shared least similarity with *E. ruminantium* and the Panola Mountain *Ehrlichia*.

**Table 2:** Percent similarities (lower-left diagonal half) and actual numbers of mismatches (upper-right diagonal half) in the *gltA* sequences (126 bp) of groups of *Ehrlichia* in Caribbean domestic ruminants and two representatives of each of the most closely related *Ehrlichia* species/strains in GenBank

	G1	G2	G3	G4	G5	UFMG	*canis*	*muris*	*chaff.*	*E.* tick	*ewing.*	*rumin.*	P-Mtn
Group1^a^		2	6	7	17	2	9	9	13	17	17	17	18
Group2	98		9	10	19	5	11	11	15	20	20	20	21
Group3	95	93		1	14	9	5	10	12	15	17	15	17
Group4	94	92	99		13	9	5	10	11–12	14	17	14	17
Group5	87	85	89	90		17	17	11	14	1	13	7	13
*E*. sp. BOV2010/UFMG-EV^b^	**98**	**96**	93	93	86		6	10	13	16–18	17	17	17
*E. canis*	93	91	**96**	**96**	86	95		11	13	14–17	15–18	14	18
*E. muris*	93	91	92	92	91	92	91		7	10–12	18–20	10	19
*E. chaffeensis*	90	88	90	91	89	90	90	95		10–15	17–18	13	19–21
*E*. sp. African ticks	86	84	88	89	**99**	86–87	87–89	90–92	88–92		13–14	5–8	13
*E. ewingii*	86	84	88	89	95	86	89	92	90	94–95		15	14–15
*E. ruminantium*	86	84	86	86	90	86	86–88	84–86	86	89–90	88		8–9
*E*. sp. P-Mtn	86	83	86	86	90	86	86	85	84–85	90	93–94	88–89	

^a^We found identical sequences in 8 (Group 1), 1 (Group 2), 3 (Group 3), 2 (Group 4) and 1 (Group 5) Caribbean domestic ruminants. Comparing these sequences with those of two representatives of the most closely related *Ehrlichia* species and strains in GenBank, the number of mismatches is shown in the upper-right diagonal half of the table and the percentage similarity is shown in the bottom-left diagonal half of the table. Matches with the highest percent similarity are shown in bold

^b^The Gene Accession numbers for representing *Ehrlichia* spp. in this table are: JN673762 and JX629807 for *E*. sp. BOV2010/UFMG-EV; AY647155 and NC_007354 for *E. canis*; NC_023063 and AF304144 for *E. muris*; AF304142 and NC_007799 for *E. chaffeensis*; AF311966 and AF311965 for *E*. sp. African ticks; DQ365879 for *E. ewingii*; NC_005259 and DQ513396 for *E. ruminantium*; DQ363995 and EU272407 for *E*. sp. P-Mtn

The 1,015 bp *gltA* sequence we obtained for *E. ovina* and deposited in GenBank (KP719095) was 99.9 % (2 mismatches) identical to that of *E. canis* from Italy (AY647155).

## Discussion

The generic *Ehrlichia* FRET-qPCR we developed proved to be both specific and sensitive in detecting *Ehrlichia* spp. in controlled experiments. In a single reaction it reliably detected the five commonly recognized *Ehrlichia* spp. we used in our experiments as well as less well characterized *Ehrlichia* which have been found in domestic ruminants and are available for study [26]. The specificity of the PCR was shown by its failure to detect representatives of the closely related *Anaplasma* genus, *A. marginale* and *A. phagocytophilum*, and the fact that all the positive reaction products had sequences that were closest to *Ehrlichia* spp. When tested against dilutions of *E. canis*, the sensitivity of the generic *Ehrlichia* FRET-qPCR was high, detecting as few as 5 copies of the *16S rRNA* gene in a reaction [27].

The *16S rRNA* gene we detected in our generic *Ehrlichia* FRET-qPCR is a common target for PCRs for *Ehrlichia* spp. as its nucleotide sequence is highly conserved in the genus. By systematically aligning the sequences of the main *Ehrlichia* spp. and closely related organisms, we were able to identify a highly conserved region of the *16S rRNA* gene against which we developed specific primers that only amplified *Ehrlichia* spp. and not organisms from related genera. Further, the region of the *16S rRNA* gene we selected for our LCRed 640 probe had nucleotide mismatches between the major *Ehrlichia* spp. which enabled us to differentiate the organisms into groups by melting point analysis (Fig. 1).

When we tested our generic *Ehrlichia* FRET-qPCR against known *Ehrlichia* spp. it detected all the organisms in a single reaction and also differentiated the species to a large extent. Using high-resolution melting point analysis we were able to clearly differentiate *E. ruminantium*, the Panola Mountain *Ehrlichia*, a group containing *E. chaffeensis* and *E. ewingii*, and a group containing *E. muris* as well as *E. canis* and organisms closely related to it. The groupings appear to be largely serendipitous, rather than of taxonomic significance, as molecular studies have shown *E. muris* is more closely related to *E. chaffeensis* than to *E. canis* [26]. Similarly, *E. ewingii* is closer to *E. canis* [27] or the Panola Mountain *Ehrlichia* [9] than to *E. chaffeensis*.

When we applied our generic *Ehrlichia* FRET-qPCR to DNA from whole blood collected from domestic ruminants from five Caribbean islands, we identified relatively high prevalences of infections (12 %) with *Ehrlichia* spp. that were not *E. ruminantium*. Of note is the fact these generic *Ehrlichia* FRET-qPCR positive animals had previously tested negative for antibodies to *E. ruminantium* in a MAP-1B ELISA [20]. This test not only detects antibodies to *E. ruminantium* [6] but also to the Panola Mountain *Ehrlichia* [9], and *E. canis* and *E. chaffeensis* [6]. It seems unlikely, then, that the animals we found positive in our generic *Ehrlichia* FRET-qPCR had been infected with these agents. While there are no data for *Ehrlichia* sp. BOV2010 and *Ehrlichia* sp. UMFG-EF, sera from animals infected with *E. ovina* do not give positive MAP-1B ELISA reactions [6] and these, or closely related organisms, seem most likely to have been detected by our generic *Ehrlichia* FRET-qPCR in the seronegative animals. Also of note is that none of the animals that had previously been found to be positive in MAP-1B ELISAs [10] were positive in our generic *Ehrlichia* FRET-qPCR. Most of these animals, however, were only very weakly positive in the MAP-1B ELISA suggesting they had residual antibody titers following clearance of infections, or that the infecting *Ehrlichia* spp. did not generate a substantial humoral response. Further studies are underway in our laboratories to clarify the position.

Melting point analysis and sequencing suggested that the *Ehrlichia* we identified with our generic *Ehrlichia* FRET-qPCR, utilizing the *16S rRNA* gene, were *E. canis* or closely related organisms. The *16S rRNA* gene is highly conserved in *E canis*, being 99.4–100 % identical between strains [28, 29], and hence a reliable way of identifying isolates. Although we sequenced only a relatively short segment of the gene (210 bp), the *Ehrlichia* spp. we identified with our generic *Ehrlichia* FRET-qPCR had 100 % homology with *E. canis* sequences in GenBank.

We found only relatively small numbers of organisms in the blood samples we studied (average copy number 231, median 9.5) with our generic *Ehrlichia* FRET-qPCR, most likely because we were detecting chronic subclinical infections but also perhaps because we were detecting infections in accidental and unsuitable hosts. The low copy numbers in our samples were also evident from the results of our *gltA* gene PCRs where we only found positive results after nesting. The *gltA* gene has also been shown to be highly conserved in *E. canis* (over 99 %) [30], but it has greater interspecies variability than the *16S rRNA* gene which might make it more useful for differentiating *Ehrlichia* species [9, 31]. The sequences we obtained for our nested *gltA*, however, were consistent with the *16S rRNA* gene findings that the organisms present in the Caribbean domestic ruminants we studied were *E. canis* or closely related species.

We would note that, because of low copy numbers in our samples, the sequences we obtained from our nested *gltA* PCR were with the internal primers and thus relatively short (126 bp). These internal primers, however, amplify a hypervariable region of the *gltA* which enables accurate discrimination of species and strains. When we compared the sequences we obtained with others in GenBank we found that, consistent with comparisons of our *16S rRNA* gene sequences, the organisms present in the Caribbean domestic ruminants were closest to *E. canis* or closely related organisms.

Although *E. canis* is best known as a very common dog pathogen around the world, including in the Caribbean [20, 32], infections have also been described in humans [33] and cats [34]. There is a growing belief that *E. canis* has a wider host range than previously thought [1, 14], and our findings are largely consistent with this idea. Of further note is that a number of *Ehrlichia* that appear to be closely related to *E. canis*, possibly even strains of this organism, have been reported in domestic ruminants. *E. ovina* (AF318946) was first recovered from a sheep in Turkey and subsequently caused illness in splenectomized Dutch sheep [4]. More recently it has been found to have an identical *16S rRNA* sequence to *E. canis* in dogs from Turkey (Kutahya strain; AY621071) [35] and Venezuela (VHE strain; AF373612) [36]. In our study, *E. ovina* had a *T*_*m*_ and *16S rRNA* sequence identical to that of the Oklahoma strain of *E. canis* (NR_118741) and of local Caribbean strains we found. Further, *E. ovina* reacted with primers for the *gltA* of *Ehrlichia* spp. and produced a 1015 bp sequence that contained only 2 mismatches with *E. canis* from Italy (AY647155). These findings provide further support for the proposal that *E. ovina* is a strain of *E. canis* [1, 34].

Recent studies have identified 3 novel cattle-related strains of *Ehrlichia*: in Canada, the *Ehrlichia* sp. BOV 2010 [13]; and in Brazil, the *Ehrlichia* sp. UFMG-EV in *Rhipicephalus microplus* hemolymph [37] and the *Ehrlichia* sp. UFMT-BV in cattle [1]. Molecular studies have shown these organisms are very similar to one another and that they probably evolved from a highly divergent and variable clade within *E. canis* [38]. The phenotypic and genotypic differences the strains have with *E. canis* have been ascribed to the organisms adapting to their new hosts, ruminants, and their new tick vectors. More detailed genomic and transmission studies might provide justification for the organism being classified as a distinct species, *E. mineirensis* [38].

## Conclusions

In conclusion, the *Ehrlichia* FRET-qPCR we developed proved sensitive and specific in detecting the most recognized *Ehrlichia* spp. of ruminants in a single reaction. Further, using melting point analysis we could differentiate the organisms into four groups comprising *E. ruminantium*; *E. chaffeensis* and *E. ewingii*; *E. canis* and closely related organisms such as *E. ovina* and *Ehrlichia* sp. BOV2010/*Ehrlichia* sp. UFMG-EV; and the Panola Mountain *Ehrlichia*. When we used the generic *Ehrlichia* FRET-qPCR on DNA from the blood of Caribbean domestic ruminants, we found a relatively high percentage (12.2 %) were positive. Melting point analysis showed the *Ehrlichia* in the Caribbean domestic ruminants were most similar to organisms in the group comprising *E. canis* and closely related species.
